# Assessment of the relationship between serum uric acid and glucose levels in healthy, prediabetic and diabetic individuals

**DOI:** 10.1186/s13098-019-0446-6

**Published:** 2019-06-21

**Authors:** Tangigul Haque, Sadaqur Rahman, Shiful Islam, Noyan Hossain Molla, Nurshad Ali

**Affiliations:** Department of Biochemistry and Molecular Biology, Shahjalal University of Science and Technology, Sylhet, 3114 Bangladesh

**Keywords:** Serum uric acid, Glucose, Diabetes, Bangladesh

## Abstract

**Background:**

In epidemiological studies, serum uric acid (SUA) has been shown to be associated with hypertension and cardiovascular disorders. However, limited studies have evaluated the relationship between SUA and glucose levels in healthy and diabetic individuals and their observed findings are inconsistent. This study aimed to examine the relationship between SUA and fasting blood glucose (FBG) levels among healthy, prediabetic and diabetic individuals in Bangladesh.

**Methods:**

In total, 310 blood samples were collected from 215 male and 95 female subjects and analyzed for FBG, SUA, and lipid levels. All participants were categorized into four quartiles based on SUA concentrations. Diabetes and prediabetes were defined as FBG level ≥ 126 mg/dL and 100–125 mg/dL, respectively. The association between SUA and diabetes was evaluated by multinomial logistic regression analysis.

**Results:**

The prediabetic and diabetic individuals had a lower mean level of SUA (338.2 ± 101.6 and 290.9 ± 98.2 µmol/L, respectively) compared to healthy (369.5 ± 110.9 µmol/L) individuals (*p *< 0.001). SUA was positively associated with BMI, TG and TC but negatively associated with FBG. The prevalence of diabetes was decreased with increasing concentration of SUA across the quartiles. In regression analysis, SUA levels were inversely associated with diabetes mellitus.

**Conclusions:**

SUA levels were high in healthy individuals but declined in prediabetic and diabetic individuals with increasing FBG concentrations. A significant inverse association was observed between the levels of SUA and diabetes in Bangladeshi adults. Further studies are needed to examine the reliability of using SUA to predict diabetes.

## Background

Uric acid in serum is the metabolic end product of the purine nucleotides, and it’s over-production and decreased excretion through kidneys lead to hyperuricemia in humans [1, 2]. The prevalence of hyperuricemia in the general population is estimated at about 10–25%. In recent decades, the prevalence of hyperuricemia has increased substantially in the world with a rising trend both in the developed and developing nations [3]. High concentration of uric acid in the blood can lead to gout and are associated with several medical conditions, including metabolic syndrome, cardiovascular diseases, diabetes and renal dysfunction [1, 3, 4]. In epidemiological studies, serum uric acid (SUA) has been shown as a risk factor for hypertension, dyslipidemia, cardiovascular and kidney diseases [5–9] however, the putative association between SUA and diabetes is not clear and the findings are controversial, and there may be sex and ethnic differences in the relationships.

Previously, some studies reported a positive association between elevated SUA and diabetes [10–13], whereas, other studies reported no correlation [14, 15], or an inverse relationship [16–20]. In healthy individuals, SUA has been reported to be positively correlated with blood glucose [19]. However, this association in non-diabetic healthy, prediabetic and diabetic individuals is not consistent. A meta-analysis reported an association between SUA and increased risk of development of type 2 diabetes (T2D) [13]. A prospective study indicated that diabetes is related to a lower risk of gout development in the UK general population [21] and Chinese population [22]. Other studies have shown a declining trend of SUA in the glycemic state [16, 19, 20] and in some cases, there was no significant association between SUA and glycemic state [14, 15]. Most of the previous studies were conducted in the population group with existing co-morbid conditions such as old age, diabetes, and who were at high risk for kidney or cardiovascular disease [6, 8, 10, 16, 23–26]. There are few studies that have evaluated the SUA levels and its relationship with blood glucose concentrations in apparently healthy adults. So, it is important to know the actual trend of SUA in healthy, prediabetic and diabetic individuals, as hyperuricemia is increasingly found to be associated with a number of modifiable risk factors contributing to cardiovascular diseases. Although there are some reports on the relationship between SUA and diabetes from different parts of the world, still there is a lack of such information for the Bangladeshi population. In this context, we aimed to assess the relationship between SUA and fasting blood glucose (FBG) levels in non-diabetic healthy, prediabetic and diabetic individuals in Bangladesh.

## Methods

### Study population

The present study consisted of both male and female participants aged above 20 years and conducted between November 2017 and October 2018. Apparently healthy individuals without having severe illness were randomly enrolled from non-academic and academic staffs and students of Shahjalal University of Science and Technology, Bangladesh. Diabetic individuals were enrolled from Sylhet Diabetic Hospital; the presence of diabetes was confirmed based on the guideline of American Diabetes Association and subjects self-reported evidence. Fasting blood samples were collected from 310 adults (215 males and 95 females) and the participants were grouped as non-diabetic healthy (n = 102), prediabetes (n = 98) and diabetes (n = 110). Pregnant women, lactating mother, individuals with having a history of anti-hyperuricemic medications intake and gout were excluded from the study. We further excluded subjects with self-reported kidney diseases, hepatic disorders and cardiovascular disease. This study was approved by the Internal Ethics Committee at the Department of Biochemistry and Molecular Biology, Shahjalal University of Science and Technology, Bangladesh. Written informed consent was obtained from each participant before inclusion in the study. All the steps of the methods section were conducted in accordance with the standard guidelines and regulations.

### General data collection

Data were collected on individual health status, anthropometric characteristics and incident of disease. Individual anthropometric data (e.g. age, gender, height and weight), food habits and lifestyle information were recorded in a brief questionnaire form followed a standard procedure described elsewhere [5, 27]. Briefly, body height was measured to the nearest 0.1 cm by height measuring tape and body weight was measured to the nearest 0.1 kg by a digital weighing machine (Beurer 700, Germany) wearing light clothes and no shoes. Body mass index (BMI) was calculated as body weight in kg divided by body height in meters squared (kg/m^2^). The accuracy of the data was confirmed by repeated measurements.

### Blood collection and laboratory measurements

After an overnight fast (10–12 h), blood samples were obtained from the participants by means of venipuncture. The samples were then put into an ice-cooled box and transported immediately to the clinical lab at the Department of Biochemistry and Molecular Biology. After centrifugation of blood samples at 3000 rpm for 15 min, the serum was isolated and stored at − 20 °C. The serum glucose concentration was measured within 3 h after of blood collection. Serum uric acid (SUA), total cholesterol (TC), triglycerides (TG), high-density lipoprotein (HDL), serum albumin and total protein were measured by colorimetric methods with a semi-auto biochemistry analyzer (Humalyzer 3000, USA). Commercially available diagnostic kits (Human Diagnostic, Germany) were used for the analysis of the above markers. All measurements were carried out in the clinical lab of the Department according to the standard manufacturer’s protocols. All laboratory tests were performed by trained graduate level students and precision of the measurements was maintained by regular method calibration with the reference standard.

### Diagnostic criteria

Diabetes was defined according to American Diabetes Association as a fasting blood plasma glucose ≥ 126 mg/dL, non-fasting plasma glucose ≥ 200 mg/dL [28], or self-reported recent use of insulin or hypoglycemic medication. Prediabetes was determined among the participants who had fasting plasma glucose level 100–125 mg/dL [28]. Non-diabetic healthy individuals (normoglycemia) was identified based on fasting plasma glucose level < 100 mg/dL and absence of the criteria that relates to prediabetes and diabetes. In present study, hyperuricemia was defined if participant having their SUA concentration was > 7.0 mg/dL (416.4 µmol/L) in men or > 6.0 mg/dL (356.9 µmol/L) in women [29, 30]. Metabolic syndrome (MetS) was identified according to the National Cholesterol Education Program-Adult Treatment Panel III (NCEP-ATP III) criteria [31]. The components were defined as following: (i) high blood pressure (BP ≥ 130/85 mmHg); (ii) waist circumference > 102 cm for men and > 88 cm for women (iii) hypertriglyceridemia (TG ≥ 150 mg/dL), (iv) low HDL-C (< 40 mg/dL for men and < 50 mg/dL for women) and (v) hyperglycaemia (100 mg/dL > FBG). Individuals with at least three of the above components were identified as having MetS. In present study, we did not have information on blood pressure and waist circumference; therefore, MetS was determined based on TG, HDL and FBG.

### Statistical analysis

Statistical data analyses were done using IBM SPSS version 23. Data are presented as mean ± SD and quartile ranges. Baseline characteristics of the participant in the groups and SUA quartiles were compared by One-way ANOVA. Chi-square test was applied to determine the differences in the percentage of individuals with hyperuricemia and MetS among the groups. Differences for the anthropometric and baseline characteristics in the gender groups were performed by independent sample t-test. Pearson’s correlation coefficient test was performed to assess the relationships of baseline variables with SUA concentrations. The association between SUA levels and diabetes was evaluated by multinomial logistic regression analysis. Diabetes was categorized as yes (presence) and no (absence), other variables were as continuous variables. In regression analysis, diabetes (yes) was taken as the dependent variable and SUA as the independent variable. SUA and other covariates were used as continuous variables in the models. We used three models in the regression analysis. Model 1 was adjusted for age (years) and gender (male and female). Model 2 was adjusted for age (years), gender (male and female), BMI (kg/m^2^), TG, TC and HDL (mg/dL). Model 3 was further adjusted for variables used in model 1 and 2 and serum albumin and total protein (g/dL). A p-value of < 0.05 was considered statistically significant.

## Results

### General characteristics of the study population

Baseline characteristics of the study subjects are presented in Table 1. Out of 310, 215 were male and 95 were female participants. The average age for all subjects was 39.6 ± 16.1 years. Participants in the diabetes group were more like to be older, overweight and obese and have higher TG and TC concentrations compared to the non-diabetic healthy and prediabetic individuals. Participants in the prediabetic and diabetic groups had a lower mean level of SUA (338.2 ± 101.6 and 290.9 ± 98.2 µmol/L, respectively) compared to the healthy (369.5 ± 110.9 µmol/L) group (*p *< 0.001). Overall, the prevalence of hyperuricemia was 18.4% with a low percentage in the diabetic group (12.7%).

**Table 1:** General characteristic of all study participants

Variables	All	Non-diabetes	Prediabetes	Diabetes	p-values
*N*	310	102	98	110	–
Sex, m/f	215/95	76/26	66/32	73/37	–
Age, year	39.6 ± 16.1	30.3 ± 13.0	39.4 ± 17.6	47.1 ± 13.1	0.000
BMI, kg/m^2^	24.6 ± 3.9	24.7 ± 4.1	23.7 ± 3.7	25.3 ± 3.8	0.045
Glucose, mg/dL	146.4 ± 77.8	89.4 ± 8.3	111.6 ± 8.2	217.4 ± 82.3	0.000
TG, mg/dL	191.5 ± 122.7	163.8 ± 85.6	160.3 ± 93.0	243.4 ± 154.2	0.000
TC, mg/dL	206.7 ± 83.1	173.7 ± 50.0	185.9 ± 51.3	254.7 ± 108.3	0.000
HDL, mg/dL	35.2 ± 15.7	32.8 ± 11.7	36.0 ± 13.7	37.0 ± 19.7	0.256
Albumin, g/dL	47.9 ± 13.1	47.4 ± 9.4	49.2 ± 13.5	47.4 ± 15.1	0.676
Total protein, g/dL	78.9 ± 26.6	80.2 ± 26.8	74.4 ± 21.3	81.2 ± 29.7	0.298
SUA, µmol/L	329.3 ± 108.1	369.5 ± 110.9	338.2 ± 101.6	290.9 ± 98.2	0.000
Hyperuricemia, n (%)	57 (18.4)	20 (19.6)	23 (23.5)	14 (12.7)	0.000^a^

Values are presented as mean ± SD

p-values are obtained from one-way ANOVA

^a^p-value is obtained from Chi square test when the percentages of hyperuricemic individuals are compared among the groups

### Baseline variables in the SUA quartiles

Table 2 presents the baseline characteristics of the study subjects in each SUA quartile. Overall, a negative association was observed for the participant’s age with increasing SUA concentration in the quartiles. A decreasing trend at the mean level of FBG level was observed across the SUA quartiles (Q1: 177.6 ± 88.2 mg/dL; Q2: 141.5 ± 72.0 mg/dL; Q3: 139.9 ± 76.5 mg/dL and Q4: 128.6 ± 65.1 mg/dL) (p < 0.01 for trend). Mean concentrations of serum TC and TG were found to be increased with the elevated levels of SUA across the quartiles (p < 0.05 for trend).

**Table 2:** Baseline characteristics of the participants according to SUA quartiles

	Q1 (≤ 249 µmol/L)	Q2 (250–321 µmol/L)	Q3 (322–387 µmol/L)	Q4 (> 387 µmol/L)	p-values for trend
*N*	77	80	79	74	–
Sex, m/f	40/37	53/27	60/19	62/12	–
Age, year	43.1 ± 12.6	42.4 ± 18.3	36.5 ± 15.0	36.2 ± 17.0	0.047
BMI, kg/m^2^	24.9 ± 4.8	25.6 ± 3.5	25.2 ± 3.6	25.8 ± 3.7	0.046
SUA, µmol/L	204.7 ± 37.0	292.1 ± 20.6	355.7 ± 18.6	477.1 ± 83.7	0.000
TG, mg/dL	170.9 ± 97.3	237.3 ± 167.9	175.8 ± 98.3	172.7 ± 86.6	0.028
TC, mg/dL	202.5 ± 69.1	202.0 ± 78.2	212.3 ± 96.2	212.1 ± 88.8	0.047
HDL, mg/dL	32.3 ± 12.6	36.7 ± 18.9	34.6 ± 15.2	36.8 ± 14.5	0.538
Albumin, g/dL	51.8 ± 18.1	45.8 ± 10.5	46.4 ± 10.1	47.6 ± 11.4	0.090
Total protein, g/dL	74.6 ± 27.6	78.6 ± 26.5	78.1 ± 18.9	84.7 ± 31.9	0.308
Glucose, mg/dL	177.6 ± 88.2	141.5 ± 72.0	139.9 ± 76.5	128.6 ± 65.1	0.008
MetS (%)	37.8	32.7	20.8	23.8	0.000^a^

Values are presented as mean ± SD

*MetS* metabolic syndrome

p-values are obtained from one-way ANOVA

^a^p-value is obtained from Chi square test when the percentages of MetS are compared among the groups

### Effect of age and gender on SUA concentrations

Male participants had a higher concentration of SUA than in the female participants in all groups. Younger participants in non-diabetic healthy group had higher levels of SUA compared to the older age participants in the prediabetic and diabetic groups (p < 0.001). In Pearson correlation coefficient test, age was negatively associated with SUA in both healthy (p < 0.01) and prediabetes (p < 0.05) groups but a positive correlation was observed with diabetes (p < 0.01) group. This association was stronger in male compared to female participants.

### Prevalence of diabetes and prediabetes in the SUA quartiles

**Fig. 1:**
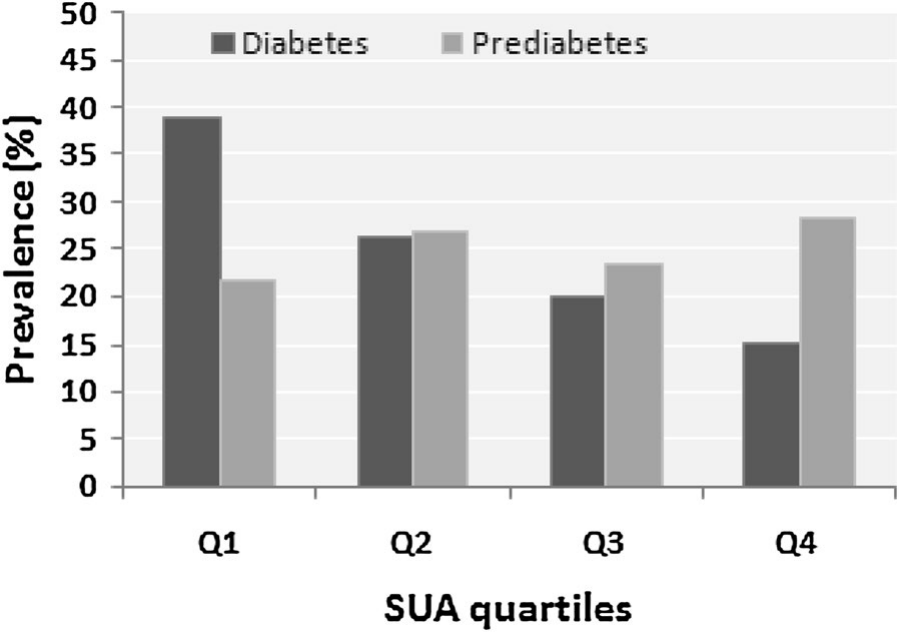
Prevalence of prediabetes and diabetes across the SUA quartiles. *p *< 0.01 for trend when diabetes prevalence is compared within the quartiles

The prevalence of diabetes was higher in the first quartile (38.7%) compared to second (26.3%), third (20%) and fourth (15%) quartile (p < 0.01 for trend) (Fig. 1). An inconsistent variation was observed on the prevalence of prediabetes across the SUA quartiles (Fig. 1). Both diabetes and prediabetes prevalence was comparatively higher in male than in the female participants.

### Association of SUA with blood glucose levels

**Fig. 2:**
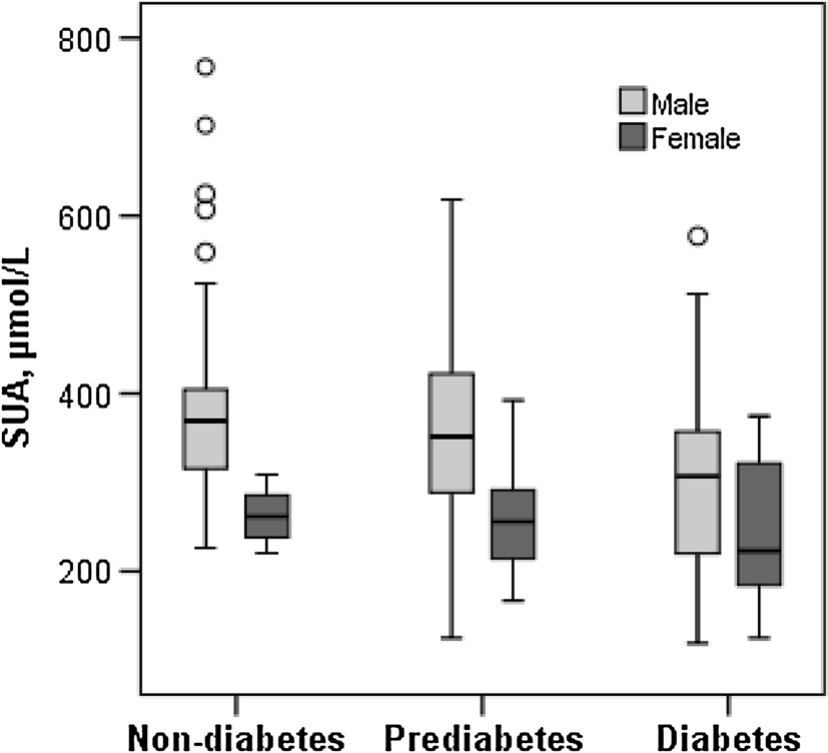
Levels of SUA in non-diabetes, prediabetes and diabetes groups by gender

Overall, a significant negative association was observed between SUA and FBG concentrations when participants in all groups are combined in Pearson’s coefficient test (p < 0.01). A significant negative relationship was also observed between SUA and FBG when individuals in non-diabetes and prediabetes groups are combined together (p < 0.01). SUA levels were decreased gradually in the prediabetes and diabetes groups compared to the non-diabetic healthy group in both genders (Fig. 2). Males had a comparatively high level of SUA than females in all groups (Fig. 2). Taking diabetes as the dependent variable and SUA as the independent variable, multinomial logistic regression was done to evaluate the association between the level of SUA and diabetes. In this regression analysis, an inverse association between SUA diabetes was observed for all participants (Table 3). In all models of regression analysis, SUA levels were significantly associated with diabetes.

**Table 3:** Multinomial logistic regression analysis to evaluate the association between SUA levels and diabetes

	*B*	SE	Wald	df	OR (95% CI)	p-value
Model 1	− 0.005	0.002	8.256	1	0.995 (0.991–0.998)	0.004
Model 2	− 0.005	0.002	5.336	1	0.995 (0.991–0.999)	0.021
Model 3	− 0.006	0.002	6.566	1	0.994 (0.990–0.999)	0.010

Dependent variable is diabetes (yes) and independent variable is SUA (µmol/L). Reference category is normal (non-diabetes). Model 1: adjusted for age (years) and gender (male and female). Model 2: model 1+ BMI (kg/m^2^), TG, TC and HDL (mg/dL). Model 3: model 2+ albumin and total protein (g/dL)

*OR* odds ratio, *CI* confidence interval, *SE* standard error

## Discussion

The present study was conducted to assess the relationship between SUA and FBG levels in non-diabetic healthy, prediabetic and diabetic individuals. To the best of our knowledge, this is the first study that shows a negative association between SUA and FBG concentration in the Bangladeshi population.

Overall, the prevalence of hyperuricemia was 18.4% with a higher percentage in the non-diabetic and prediabetic groups compared to the diabetic group. Younger participants in the non-diabetic group had a higher level of SUA compared to older age participants in the prediabetic and diabetic groups (p < 0.001). In Pearson correlation coefficient test, age was negatively associated with SUA concentrations in both healthy (p < 0.01) and prediabetes (p < 0.05) groups but a positive correlation was observed with diabetes (p < 0.01) group. This association was stronger in male compared to female participants. This higher level of SUA in younger individuals support the hypothesis that SUA might be involved in the early stages of metabolic imbalance leading to prediabetes and to a lesser extent in the advanced stages when diabetes is diagnosed [23].

Baseline characteristics of the study subjects revealed that male participants often had a higher level of SUA compared to female individuals irrespective of glycemic status. This finding is in line with the previous studies that conducted to evaluate the impact of sex on uric acid concentrations [26] and another study that assessed the relationship between SUA levels and glycemic parameters among Indian adults [19]. Moreover, in diabetic individuals, SUA levels were significantly lower than in the healthy and prediabetic individuals and showed an inverse association with FBG concentration. This is an agreement with previous studies [16, 17, 19], where a decreasing trend of SUA was observed with increasing blood glucose concentration. In multinomial logistic regression analysis, SUA was inversely associated with diabetes. This association was significant in all regression models when covariates are adjusted in model 1 (age and sex), model 2 (model 1+ BMI, TG and TC) and in model 3 (model 1 and 2+ serum albumin and total protein).

Some studies have reported a positive association between SUA and diabetes [10–13], whereas a follow-up study on Japanese individuals for 16 years, uric acid was found not to be correlated with a statistically significant increase in the risk of T2D [14]. In a recent study, no significant correlation was found between SUA and FBG in diabetic individuals in India [15]. Inverse correlation or decreased SUA concentration in diabetic individuals has been reported in other studies. For instance, a negative association between SUA and FBG has been reported in Austrian men [32]. Another study conducted on participants of the National Health and Nutrition Examination Survey indicated that SUA levels were negatively associated with T2D [16]. Such a negative association between SUA and diabetes has also been reported in other studies [17–20] that supports the present study findings. Maybe there were some reasons for the observed positive association between SUA and diabetes in previous studies. For example, in previous studies, the association between SUA and diabetes mellitus were limited to racial/ethnic groups and gender and the findings were not consistent. Moreover, many of these studies were limited by small sample sizes, including either male or female participants, aged participants, or participants were selected from a selected group other than the more general population and lack of confounding variables. We assume that besides the known associated variables, individual food habits, lifestyle, genetic and environmental factors may also have an influence on the association between SUA and diabetes in the different population groups.

A plausible mechanism for the observed findings of the negative relationship between SUA and diabetes may be related to the inhibition of uric acid reabsorption in the proximal tubule of kidney by high glucose concentrations in diabetic individuals [33, 34]. Studies demonstrated that the net concentration of uric acid in serum depends on its synthesis, secretion and reabsorption in the body [35, 36]. The low concentration of uric acid in serum might be the results of the uricosuric effect of glucose on uric acid which may influence to increase the excretion and decrease reabsorption of uric acid from the kidney [24]. About 70% of the uric acid is excreted through kidney [37]. The exact mechanism is not clear but the proposed mechanism is that the 100% of uric acid is filtered in the glomerulus to the renal tubules with about 80% filtered load reabsorbed [38]. A major percentage of uric acid is reabsorbed by proximal tubules using urate or anion exchanger and a voltage sensitive urate channels [25, 39]. The filtered blood glucose is also reabsorbed in the proximal renal tubes. As both blood glucose and uric acid are reabsorbed at the same site in the kidney, so, blood glucose may affect the uric acid reabsorption [20, 40]. Uric acid is transported by GLUT9 from lumen to proximal tubules and its reabsorption may be affected by several inorganic and organic ions and glucose which results in decline reabsorption and increase excretion of uric acid [41].

It has been demonstrated that type 2 diabetes is associated with oxidative stress and increased free radical formation [42, 43]. Oxidative stress causes the reduction of the antioxidant levels in the body [44]. This may explain the reduction of SUA in present investigation as SUA is considered as one of the total antioxidant compound present in the body [45]. The inverse relationship observed in the present study between SUA and FBG may reflect the role of hyperglycemia in the genesis of oxidative stress in diabetic individuals. The main strength of the present study includes its population-based nature, the inclusion of healthy, prediabetic and diabetic individuals, and the availability of some useful data on potential confounders for multivariable adjustment. Furthermore, a rigorous methodology was followed during data collection. The major limitation of the study is the cross-sectional nature of the data which may preclude the findings regarding the temporal nature of the relationship between SUA and diabetes. Therefore, even though there were some independent traditional confounders such as age, BMI, TC and TG, but it was difficult to confirm the cause-effect relationship. Other limitations included, we did not have information on glycemic control (HbA1c) and levels of urinary uric acid of the prediabetic and diabetic individuals, and therefore our results indicate a hypothesis other than a conviction. Moreover, we did not collect information on antidiabetic medications. Taken into account the inconsistent findings in recent and previous studies, it is rather difficult to determine SUA levels as a risk factor for diabetes mellitus. However, present study findings are worthy as a reference for future research.

## Conclusions

SUA levels were higher in non-diabetic individuals, but a decreasing trend was observed in prediabetic and diabetic individuals. This finding supports the hypothesis that SUA might be involved in the early stages of metabolic imbalance leading to prediabetes and to a lesser extent in the advanced stages of diabetes is diagnosed. So, SUA might be a determinant in altered glucose metabolism but not a potential predictor of diabetes in the Bangladeshi population. Further studies with large sample size are needed to examine the reliability of using SUA to predict diabetes.

## Data Availability

The datasets used and analyzed during the present study are available from the corresponding author on reasonable request.

## Abbreviations

SUA: serum uric acid
T2D: type 2 diabetes
FBG: fasting blood glucose
BMI: body mass index
TG: triglycerides
TC: total cholesterol
HDL: high-density lipoprotein
